# Psychological impact of the COVID‐19 pandemic on hospital workers in Kobe: A cross‐sectional survey

**DOI:** 10.1002/pcn5.8

**Published:** 2022-04-27

**Authors:** Haruko Fukushima, Hissei Imai, Chisato Miyakoshi, Hiroyuki Miyai, Kyohei Otani, Shinsuke Aoyama, Kunitaka Matsuishi

**Affiliations:** ^1^ Department of Psychiatry Kobe City Medical Center General Hospital Kobe Japan; ^2^ Health Promotion and Human Behavior, Graduate School of Medicine/School of Public Health Kyoto University, Kyoto, and Ohashi Clinic Kyoto Japan; ^3^ Department of Research Support, Center for Clinical Research and Innovation Kobe City Medical Center General Hospital Kobe Japan; ^4^ Department of Psychiatry Kobe University Graduate School of Medicine Kobe Japan

**Keywords:** COVID‐19 pandemic, health‐care workers, mental health, psychological distress, questionnaire

## Abstract

**Aim:**

Many health‐care workers exposed to coronavirus disease 2019 (COVID‐19) are psychologically distressed. This study aimed to investigate the psychological impact of the COVID‐19 pandemic on hospital workers under the emergency declaration in Japan.

**Methods:**

This cross‐sectional, survey‐based study collected sociodemographic data and responses to 19 stress‐related questions and the Impact of Event Scale‐Revised (IES‐R), which measures post‐traumatic stress disorder (PTSD) symptoms, from all 3217 staff members at Kobe City Medical Center General Hospital from April 16, 2020 to June 8, 2020. Exploratory factor analysis was applied to the 19 stress‐related questions. Multiple regression models were used to evaluate the association of personal characteristics with each score of the four factors and the IES‐R.

**Results:**

We received 951 valid responses; 640 of these were by females, and 311 were by respondents aged in their 20s. Nurses accounted for the largest percentage of the job category. Women, those aged in their 30s–50s, nurses, and frontline workers had a high risk of experiencing stress. The prevalence of stress (IES‐R ≥ 25) was 16.7%. The psychological impact was significantly greater for those aged in their 30s–50s and those who were not medical doctors.

**Conclusions:**

This is the first study to examine the stress of hospital workers, as measured by the IES‐R, under the emergency declaration in Japan. It showed that women, those aged in their 30s–50s, nurses, and frontline workers have a high risk of experiencing stress. Health and medical institutions should pay particular attention to the physical and psychological health of these staff members.

## Graphical abstract

This is the first study to examine the stress of hospital workers, as measured by the Impact of Event Scale‐Revised, under the emergency declaration in Japan. It showed that women, those aged in their 30s–50s, nurses, and frontline workers have a high risk of experiencing stress. Health and medical institutions should pay particular attention to the physical and psychological health of these staff members.

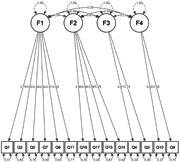

## INTRODUCTION

In December 2019, a new infectious disease outbreak was reported in Wuhan, China^1^; this was designated as coronavirus disease 2019 (COVID‐19).^2^ The World Health Organization declared COVID‐19 a pandemic on March 11, 2020. A previous study from Wuhan showed how this unprecedented situation impacted the mental health of frontline hospital workers, who reported psychological problems, such as anxiety, depressive symptoms, anger, and fear.^3^ Tackling the mental health of hospital workers during this pandemic is essential and will strengthen the capacity of health‐care systems.^4^

Previous studies have reported on hospital workers' mental health as impacted by infections, such as the 2003 severe acute respiratory syndrome (SARS),^5^, ^6^ 2009 (H1N1) influenza,^7^, ^8^ and 2015 Middle East respiratory syndrome (MERS).^9^ Mental health outcomes and associated factors among health‐care workers related to COVID‐19 have already been reported in many counties worldwide.^10^, ^11^, ^12^, ^13^, ^14^ In a meta‐analysis by Serrano‐Ripoll et al., sociodemographic factors (younger age and female gender), social factors (lack of social support and stigmatization), and occupational factors (working in high‐risk environments, specific occupational roles, and less specialized training and job experience) were identified as factors associated with the likelihood of developing psychological problems.^15^

In these systematic reviews, however, there are no reports related to COVID‐19 in Japan.^15^, ^16^ We have previously reported on the psychological impact of the 2009 (H1N1) pandemic on hospital workers^7^, ^8^ and this study extends our work by examining whether COVID‐19‐related work is associated with mental health problems in Japan. The current study was initiated on April 16, 2020, after the first state of emergency was declared on April 7, 2020. During this time, the first number of infections had peaked, and the entire country was extremely tense. This survey is the first to be conducted under Japan's declared state of emergency to determine the stress status of hospital workers using the Impact of Event Scale‐Revised (IES‐R). The IES‐R measures PTSD symptoms in survivorship after an event.

On March 3, 2020, Kobe City Medical Center General Hospital (KCGH) admitted its first COVID‐19‐infected patient in Kobe, and by the end of October 2021, 1036 patients with severe COVID‐19 had been admitted. At the beginning of the outbreak, in April 2020, nosocomial infections occurred among seven inpatients and 29 staff members, and 349 employees were requested to standby at home in quarantine to prevent the spread of infection. We believe that hospital workers experienced more severe physical and psychological stress than ever before.

To clarify the impact of the COVID‐19 pandemic on hospital workers, we distributed questionnaires to staff members working in a designated medical institution for COVID‐19 in Kobe, Japan. We then investigated the psychological impact of the COVID‐19 pandemic on hospital workers and how it varied by the characteristics of gender, age, job, and work environment.

## METHODS

### Study setting and participants

This was an observational, hospital‐based study. In April 2020, soon after the pandemic began in Kobe City, Japan, a questionnaire comprising questions on sociodemographic characteristics, 19 stress‐related questions, and the IES‐R were distributed to all 3217 employees at KCGH. The 289 employees on standby at home were sent the questionnaire by mail. Data were collected from April 16, 2020 to June 8, 2020. Written informed consent was obtained, but participants could also choose to remain anonymous. In accordance with the International Ethical Guidelines for Health‐related Research Involving Humans, all employees were notified of the research information and purpose; the disclosure document was sent via email and the employees were offered the opportunity to refuse. Questionnaires were completed by 1111 employees. Of these responses, 160 contained at least one missing answer, leaving 951 questionnaires (response rate: 29.6%) for analysis. The characteristics of the valid respondents are listed in Table 1. The study protocol was approved by the Ethics Committee of KCGH (no. zn200726).

**TABLE 1:** The characteristics of the valid respondents

	Valid respondents (*n*)
Asked to standby at home	
No	769
Yes	182
Gender	
Male	311
Female	640
Age group (years)	
20–29	311
30–39	244
40–49	223
50–59	129
60+	44
Job	
Medical doctor	157
Nurse	343
Others	451

*Note*: Job classified as medical doctor, nurse, or others (radiologic technologists, clinical laboratory technicians, pharmacists, dieticians, social workers, physical therapists, occupational therapists and speech therapists, biomedical equipment technicians, office workers, clinical clerks, guards, and janitors).

### Procedure

Starting on April 16, 2020, self‐administered paper questionnaires were handed to all employees directly or placed in their mailboxes. Additionally, an e‐questionnaire, created using Microsoft Forms, was shared through email. Questionnaires were collected until June 8, 2020, the estimated peak of the first wave of the COVID‐19 pandemic in Kobe City.

### Content of questionnaire

The questionnaire explained the study's purpose, which was to examine the stress experienced by hospital workers during the COVID‐19 pandemic. It comprised items covering sociodemographic characteristics, stress‐related questions associated with the COVID‐19 outbreak, and the IES‐R.

Personal characteristics included gender, age group, job, and work environment during the COVID‐19 pandemic. The respondents were asked if they had experienced the Great Hanshin‐Awaji Earthquake and whether they had participated in a Disaster Medical Assistance Team (DMAT). Work environment was categorized into frontliner (a respondent working in the ward for COVID‐19 infection and the fever consultation center) and non‐frontliner (a respondent working in any other place).

There were 19 questions related to stress (Table 2). The respondents indicated using a four‐point Likert scale how often during the pandemic they experienced the conditions covered by these items. The 19 items used in our study were based on similar items in a stress questionnaire used to study an influenza pandemic (H1N1) 2009.^7^, ^8^

**TABLE 2:** Stress‐related questions associated with the coronavirus disease 2019 (COVID‐19) outbreak

		0 *Never*	1 *Rarely*	2 *Sometimes*	3 *Always*
1	I felt anxious about being infected.				
2	I felt anxious about infecting my family.				
3	I felt burdened by the increased quantity of work.				
4	I felt burdened by the changed quality of work.				
5	I felt anxious about being infected during commuting.				
6	I felt lacking in knowledge about prevention and protection.				
7	I felt lacking in knowledge about infectiosity and virulence.				
8	I felt avoided by others.				
9	I felt protected by my country or local government.				
10	I felt protected by my hospital.				
11	I felt anxious about compensation in the case of being infected.				
12	I felt hesitance to work.				
13	I felt isolated.				
14	I felt elevated mood.				
15	I had insomnia.				
16	I was exhausted physically.				
17	I was exhausted mentally.				
18	I felt motivated to work.				
19	I felt I had no choice but to work due to obligation.				

The IES‐R is a self‐report measure of current subjective distress in response to a specific traumatic event. This 22‐item scale comprises three subscales representative of the major symptom clusters of post‐traumatic stress: intrusion, avoidance, and hyperarousal.^17^ The respondent is asked to report the degree of distress experienced for an item in the past 7 days. The five points on the scale are: 0 (*not at all*), 1 (*a little bit*), 2 (*moderately*), 3 (*quite a bit*), and 4 (*extremely*). The reliability and validity of the Japanese version of the IES‐R have been verified. A cut‐off score of 24/25 was used to define post‐traumatic stress disorder (PTSD) of clinical concern.^18^

### Data analysis

The characteristics of the participants were summarized as numbers and percentages for categorical variables, and as mean and standard deviation (SD) for continuous variables.

In our previous study, during the H1N1 influenza pandemic, we performed an exploratory factor analysis and identified four factors for evaluation (anxiety about infection, exhaustion, workload, and feeling of being protected) using a stress‐related questionnaire survey among hospital workers.^7^, ^8^ In the present study, confirmatory factor analysis (CFA) was conducted to confirm the same four‐factor structure tested by the stress‐related survey among the employees engaged in providing health‐care services during the COVID‐19 pandemic. The robust maximum likelihood estimator was used as the data were not normally distributed. Model fitting was assessed using the following indices with their respective cut‐off values: goodness of fit index (GFI) >0.9, adjusted GFI (AGFI) >0.9, comparative fit index (CFI) >0.9, and root‐mean‐square error of approximation (RMSEA) ≦0.08.

The total score of questionnaire items for each of the four factors was calculated. Each score of each factor and the IES‐R were compared between strata of each personal characteristic using either Student's *t*‐test or analysis of variance. We also evaluated the association of personal characteristics with each score and the IES‐R using multiple linear regression models. Participants with missing data were excluded from the regression analyses. The association between participants' characteristics and psychological impact (IES‐R) was tested using Fisher's exact test, and a two‐sided exact *p* value was reported. Two‐sided *p*‐values <0.05 were considered statistically significant.

All analyses were performed using R statistical software (Version 4.1.0). For the CFA, the *lavaan* package (Version 0.6‐8) was used.

## RESULTS

**FIGURE 1:**
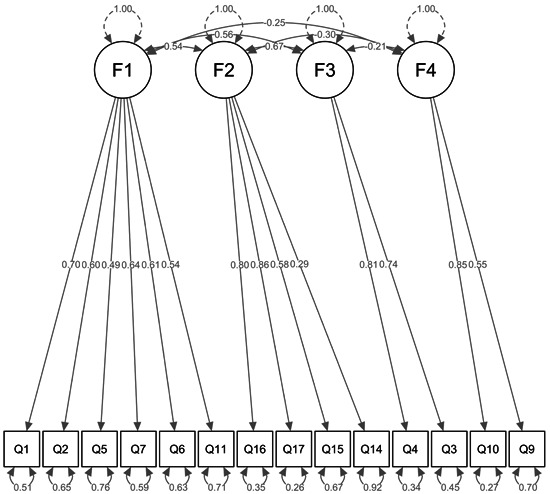
The path diagram of the fitted model with standardized estimates of factor correlation, factor loading, and variance for each item.

A total of 1111 employees completed the questionnaire. CFA was conducted using data from 1111 surveys. In this model, Factor 1 consisted of Items 1, 2, 5, 6, 7, and 11, representing “anxiety about infection.” Factor 2 included Items 14, 15, 16, and 17, indicating “exhaustion.” Factor 3 consisted of Items 3 and 4, which represented “workload.” Factor 4, which indicated “feeling of being protected,” was based on Items 9 and 10. Figure 1 presents the path diagram of the fitted model. Model fitting was acceptable: *χ* ^2^ (*df*) = 394.0 (71), *p* < 0.001; GFI = 0.942; AGFI = 0.914; CFI = 0.911; RMSEA (90% confidence interval) = 0.064 (0.058–0.070).

There were moderate correlations between each score of the four factors and the IES‐R score (Pearson product–moment correlation). Factor 1 (anxiety about infection) *γ* = 0.43, *γ* ^2^ = 0.18, *p* < 0.001, *n* = 951; Factor 2 (exhaustion) *γ* = 0.57, *γ* ^2^ = 0.32, *p* < 0.001, *n* = 951; Factor 3 (workload) *γ* = 0.31, *γ* ^2^ = 0.10, *p* < 0.001, *n* = 951; Factor 4 (feeling of being protected) *γ* = −0.19, *γ* ^2^ = 0.04, *p* < 0.001, *n* = 951. These results indicate some of the validity of the stress‐related questions associated with the COVID‐19 outbreak conducted in this study.

### Regression analysis

The data of 951 (29.6%) participants were included in the regression analysis. Table 3 lists the estimated associations of the sociodemographic characteristics with the total score for each of the four factors and the IES‐R. The independent variables were gender, age group, job, being asked to standby at home, exposure to COVID‐19, experience of the Great Hanshi‐Awaji Earthquake, and experience of engagement in DMAT. The dependent variables were Factors 1, 2, 3, and 4, and the IES‐R score.

**TABLE 3:** Regression analysis

			F1 (anxiety about infection): Q1, Q2, Q5, Q7, Q6, Q11					F2 (exhaustion): Q16, Q17, Q15, Q14				
					Univariable	Multivariable				Univariable	Multivariable	
	*N*	(%)	Mean	(SD)	*p*‐value	*β*	*p*‐value	Mean	(SD)	*p*‐value	*β*	*p*‐value
Asked to standby at home												
No	769	(80.9)	9.79	(3.04)	<0.001	–		4.14	(2.54)	0.219	–	
Yes	182	(19.1)	11.26	(2.66)		0.980	<0.001	4.40	(2.35)		−0.060	0.795
Sex												
Male	311	(32.7)	9.14	(3.18)	<0.001	–		3.82	(2.56)	0.001	–	
Female	640	(67.3)	10.52	(2.85)		0.690	0.003	4.38	(2.46)		0.300	0.128
Age (years)												
20–29	311	(32.7)	9.93	(3.13)	0.176	–		3.66	(2.39)	<0.001	–	
30–39	244	(26)	10.04	(3.14)		0.240	0.329	4.41	(2.59)		0.770	<0.001
40–49	223	(23.4)	10.35	(2.94)		0.550	0.034	4.61	(2.59)		0.970	<0.001
50–59	129	(13.6)	10.26	(2.57)		0.410	0.233	4.60	(2.22)		0.800	0.007
60+	44	(4.6)	9.25	(3.26)		−0.270	0.587	3.50	(2.54)		−0.170	0.688
Job												
Medical doctor	157	(16.5)	8.18	(2.9)	<0.001	–		3.50	(2.36)	<0.001	–	
Nurse	343	(36.1)	10.79	(2.58)		1.800	<0.001	4.46	(2.37)		0.760	0.007
Others	451	(47.4)	10.18	(3.11)		1.770	<0.001	4.23	(2.62)		0.620	0.009
Exposure to COVID‐19												
Non‐frontliner	717	(75.4)	9.97	(3.04)	0.058	–		4.12	(2.52)	0.124	–	
Frontliner	234	(24.6)	10.40	(2.98)		0.270	0.242	4.41	(2.46)		0.400	0.043
Experience of the Great Hanshin‐Awaji Earthquake												
No	780	(82)	10.02	(3.13)	0.256	–		4.09	(2.52)	0.004	–	
Yes	171	(18)	10.31	(2.52)		0.090	0.744	4.69	(2.42)		0.430	0.085
Experience of engagement in DMAT												
No	919	(96.6)	10.10	(3.03)	0.119	–		4.21	(2.51)	0.342	–	
Yes	32	(3.4)	9.25	(2.93)		−0.190	0.716	3.78	(2.52)		−0.310	0.500

	F3 (workload): Q4, Q3					F4 (feeling of being protected): Q10, Q9					IES‐R				
			Univariable	Multivariable				Univariable	Multivariable				Univariable	Multivariable	
	Mean	(SD)	*p*‐value	*β*	*p*‐value	Mean	(SD)	*p*‐value	β	*p*‐value	Mean	(SD)	*p*‐value	*β*	*p*‐value
Asked to standby at home															
No	2.22	(1.47)	<0.001	–		2.03	(1.22)	0.481	–		12.32	(13.17)	0.065	–	
Yes	3.03	(1.54)		0.32	0.018	2.10	(1.2)		0.23	0.048	14.35	(13.99)		1.61	0.194
Sex															
Male	2.17	(1.45)	0.003	–		2.24	(1.22)	<0.001	–		12.12	(13.46)	0.337	–	
Female	2.48	(1.54)		−0.01	0.897	1.95	(1.2)		−0.24	0.013	13.00	(13.29)		−0.4	0.707
Age (years)															
20–29	2.07	(1.51)	<0.001	–		2.11	(1.26)	0.364	–		10.80	(12.93)	0.022	–	
30–39	2.45	(1.6)		0.44	<0.001	1.91	(1.18)		−0.23	0.028	13.38	(13.13)		2.7	0.018
40–49	2.70	(1.48)		0.76	<0.001	2.08	(1.18)		−0.05	0.661	13.83	(13.86)		3.37	0.005
50–59	2.50	(1.37)		0.54	0.002	2.08	(1.27)		−0.05	0.738	14.55	(13.74)		3.96	0.012
60+	2.14	(1.36)		0.18	0.473	2.09	(1.21)		−0.10	0.644	11.48	(12.48)		1.42	0.539
Job															
Medical doctor	2.06	(1.49)	<0.001	–		2.34	(1.18)	0.003	–		8.46	(9.8)	<0.001	–	
Nurse	2.88	(1.4)		0.72	<0.001	1.95	(1.21)		−0.32	0.024	13.06	(12.85)		4.38	0.004
Others	2.10	(1.52)		0.12	0.397	2.02	(1.22)		−0.27	0.022	13.93	(14.47)		5.65	0.000
Exposure to COVID‐19															
Non‐frontliner	2.19	(1.47)	<0.001	–		2.06	(1.21)	0.454	–		12.43	(13.22)	0.249	–	
Frontliner	2.93	(1.53)		0.59	<0.001	2.00	(1.22)		−0.13	0.179	13.59	(13.74)		1.53	0.153
Experience of the Great Hanshin‐Awaji Earthquake															
No	2.33	(1.55)	0.031	–		2.05	(1.2)	0.830	–		12.46	(13.29)	0.208	–	
Yes	2.60	(1.34)		0.12	0.396	2.03	(1.27)		0.00	0.993	13.88	(13.59)		−0.07	0.958
Experience of engagement in DMAT															
No	2.37	(1.53)	0.555	–		2.04	(1.21)	0.606	–		12.77	(13.34)	0.503	–	
Yes	2.53	(1.08)		0.13	0.634	2.16	(1.27)		−0.02	0.947	11.16	(13.76)		−1.09	0.656

Abbreviations: COVID‐19, coronavirus disease 2019; DMAT, disaster medical assistance team.

For Factor 1, “anxiety about infection,” workers who were asked to standby at home had more anxiety than workers who were not asked to standby at home (*β* = 0.98, *p* < 0.001). Regarding gender, females reported higher levels of anxiety than males (*β* = 0.69, *p* = 0.003). Workers in their 40s experienced higher levels of anxiety than workers in their 20s (*β* = 0.55, *p* = 0.034). Related to job category, nurses and others had higher levels of anxiety about infection than medical doctors (nurses: *β* = 1.80, *p* < 0.001; others: *β* = 1.77, *p* < 0.001).

For Factor 2, “exhaustion,” workers in their 30s, 40s, and 50s reported feeling more exhaustion than workers in their 20s (30s: *β* = 0.77, *p* < 0.001; 40s: *β* = 0.97, *p* < 0.001; 50s: *β* = 0.80, *p* = 0.007). Related to job category, nurses and others felt more exhausted than medical doctors (nurses: *β* = 0.76, *p* = 0.007; others: *β* = 0.62, *p* = 0.009). Regarding exposure to COVID‐19, frontliners felt more exhausted than non‐frontliners (*β* = 0.400, *p* = 0.043).

For Factor 3, “workload,” workers who were asked to standby at home reported higher workload than workers who were not asked to standby at home (*β* = 0.32, *p* < 0.018). Workers in their 30s, 40s, and 50s reported more demanding workload than those in their 20s (30s: *β* = 0.44, *p* < 0.001; 40s: *β* = 0.76, *p* < 0.001; 50s: *β* = 0.54, *p* = 0.002). Nurses reported that they had a greater workload than medical doctors (*β* = 0.72, *p* < 0.001). Related to exposure to COVID‐19, frontliners felt that they had a higher workload than non‐frontliners (*β* = 0.59, *p* < 0.001).

For Factor 4, “feeling of being protected,” workers who were asked to standby at home had a stronger feeling of being protected than workers not asked to standby at home (*β* = 0.23, *p* = 0.048). Regarding gender, females felt less well‐protected than did males (*β* = −0.24, *p* = 0.013). Workers in their 30s had less of a sense of being protected than those in their 20s (*β* = −0.23, *p* = 0.028). Regarding their job, nurses and others reported feeling less well‐protected than did medical doctors (nurses: *β* = −0.32, *p* = 0.024; others: *β* = −0.27, *p* = 0.022).

The mean (SD) total score on the IES‐R in 951 participants was 12.7 (13.3), with a range of 0–73. Notably, 159 (16.7%) respondents screened positive on clinical concern for PTSD (Table 4). The results of detailed demographic data for the severe group who were suspected of having PTSD were described in Table 5. The psychological impact was significantly related to the job (*p* = 0.006). In regression analysis, the total IES‐R scores varied by age and job. The total IES‐R scores of workers in their 30s, 40s, and 50s were higher than those of workers in their 20s (30s: *β* = 2.7, *p* = 0.018; 40s: *β* = 3.37, *p* = 0.005; 50s: *β* = 3.96, *p* = 0.012). Examined by job category, the total IES‐R score of nurses and others was higher than that of medical doctors (nurses: *β* = 4.38, *p* = 0.004; others: *β* = 5.65, *p* = 0.000).

**TABLE 4:** Psychological impact of the pandemic among study participants

IES‐R	Frequency (%)
Normal (0–24)	792 (83.3)
PTSD of clinical concern (≥25)	159 (16.7)

Abbreviations: IES‐R, Impact of Event Scale‐Revised; PTSD, post‐traumatic stress disorder.

**TABLE 5:** Psychological impact of the pandemic by participants characteristics

Parameters	Normal (0–24)	PTSD of clinical concern (≥25)		
Frequency (%)	Frequency (%)	Frequency (%)	Total	*p*‐value*
Asked to standby at home				
No	642 (83.5)	127 (16.5)	769	0.729
Yes	150 (82.4)	32 (17.6)	182	
Gender				
Male	255 (82.0)	56 (18.0)	311	0.458
Female	537 (83.9)	103 (16.1)	640	
Age (years)				
20–29	266 (85.5)	45 (14.5)	311	0.175
30–39	200 (82.0)	44 (18.0)	244	
40–49	186 (83.4)	37 (16.6)	223	
50–59	100 (77.5)	29 (22.5)	129	
60+	40 (90.9)	4 (9.1)	44	
Job				
MD	143 (91.1)	14 (8.9)	157	0.006
Nurse	288 (84.0)	55 (16.0)	343	
Others	361 (80.0)	90 (20.0)	451	
Exposure to COVID‐19				
Non‐frontliner	603 (84.1)	114 (15.9)	717	0.236
Frontliner	189 (80.8)	45 (19.2)	234	

Abbreviations: COVID‐19, coronavirus disease 2019; PTSD, post‐traumatic stress disorder.

* Fisher's exact test, two‐sided.

In regression analysis, we included the experience of the great Hanshin‐Awaji earthquake and experience of engagement of DMAT as independent variables. We investigated whether the experience of the Great Hanshin‐Awaji Earthquake would be a vulnerability factor in re‐experiencing trauma, and whether the experience of DMAT participation would be a protective factor through prior education. Regression analysis showed that these experiences did not influence the total score for each of the four factors and the IES‐R.

## DISCUSSION

This is the first study examining stress of hospital workers, as measured by the IES‐R, under the emergency declaration in Japan. The study identified that among health‐care workers, women, those in their 30s–50s, nurses, and frontline workers faced multiple high‐risk factors while treating patients with COVID‐19.

### Stress and quarantine

At the beginning of the COVID‐19 outbreak, nosocomial infections occurred, and 349 employees were required to standby at home to prevent the spread of infection. Workers who had to be quarantined felt higher levels of “anxiety about infection” and a higher “workload” than those who were not quarantined. However, quarantined workers also had a stronger “feeling of being protected” than nonquarantined workers. Continuous communication between health‐care workers and managers, including the provision of up‐to‐date facts about the progression of the outbreak, conveys institutional support. Similarly, it is essential that managers take steps to mitigate feelings of social isolation and stigmatization, especially among quarantined hospital health‐care workers.^15^

### Stress and gender

In our study, females experienced higher levels of anxiety and felt less well‐protected than did males. In contrast, “exhaustion,” “workload,” and the IES‐R scores did not significantly differ between genders. Similar results have been reported previously. Female gender has been consistently associated with higher levels of stress^19^, ^20^, ^21^ and anxiety,^10^, ^20^, ^21^, ^22^, ^23^, ^24^, ^25^ whereas no consistent association has been found with PTSD.^15^ Women were more prone to anxiety and stress, and seemed to require more attentive care.

### Stress and age

In this study, “anxiety about infection” was stronger among workers in their 40s than those in their 20s. This could be because they have anxiety about themselves and their family members being infected, echoing results of studies on influenza pandemics in the United States.^26^ Stress about bringing the virus home and passing it on to family members persists.

Hospital workers in their 30s, 40s, and 50s were more exhausted and reported that they had a greater workload than workers in their 20s. In terms of sociodemographic factors, younger age is a risk factor for burnout.^27^ Our results, however, showed that older workers had greater risk for exhaustion during the emergency declaration. Another study in Japan also showed that older workers experienced more general distress than workers in their 20s.^28^ The COVID‐19 pandemic was more prolonged than the events addressed in previous studies, which may have affected the results.

### Stress and profession

Examined by job category, nurses, and others were significantly more anxious about infection and becoming exhausted, but they perceived receiving less protection than did medical doctors. Reported “workload” was significantly higher for nurses than medical doctors. Moreover, the total IES‐R score was significantly higher for nurses than medical doctors. Similar results were reported in studies of the 2003 SARS outbreak^5^, ^29^ and the 2009 influenza pandemic (H1N1) in Japan.^8^ Another study of the COVID‐19 pandemic yielded similar results.^10^ Nurses are more likely to develop PTSD,^30^ anxiety,^31^ stress,^32^ and burnout.^33^ The amount of time spent with infectious patients may explain the difference in job effects. At our hospital, information on the COVID‐19 pandemic was initially sent to hospital workers via internal email. Since only the medical doctors and managers of each department had internal email addresses, nurses and other staff may have received less information. Sharing exact information could have reduced stress and provided a favorable work environment.^8^ Based on the findings of this study, starting in July 2020, we shared the information on the cover of the electronic medical records system to enable all staff members to access it equally. It is necessary to examine whether this method has led to improvements.

### Stress and place of posting

Hospital workers in high‐risk environments (frontliners) experienced significantly higher levels of “exhaustion” and a higher “workload” than workers in low‐risk work environments (non‐frontliners). A systematic review by Serrano‐Ripoll et al. found that working in a high‐risk environment was associated with a variety of mental health problems.^15^ Prevalence of stress (IES‐R ≥ 25) was 16.7% in our study. A previous psychological survey of Chinese health‐care workers from January 29, 2020 to February 3, 2020, involving 34 hospitals in China showed that 35% of health‐care workers reported moderate to severe levels of stress (IES‐R ≥ 26).^10^ However, a study conducted from February 19 to March 13, 2020 among health‐care workers during Singapore's COVID‐19 outbreak showed a lower prevalence (7.7%) of stress (IES‐R ≥ 24) compared to that in our study,^34^ a difference that may be related to the period and place that posed maximal stress.

The “feeling of being protected” factor did not significantly differ between work environments. The total score for the “feeling of being protected” factor was low for workers in both high‐ and low‐risk areas. This could be because during the COVID‐19 pandemic in Japan, especially in the latter half of April and May 2020, national and local governments enacted infection‐control activities, but did not provide hospital workers with information about protection against or compensation for COVID‐19 infection acquired in the course of hospital duties. The supply of protective equipment was inadequate. Some staff required alternative accommodation to reduce the risk of infecting their families. In preparation for a pandemic, some studies have emphasized the need for communities and employers to take all reasonable precautions to prevent illness among health‐care providers, as well as to provide reliable compensation if workers become ill while carrying out required duties.^35^, ^36^, ^37^

Motivated by our study results, our hospital's director began the practice of sending regular messages of comfort, encouragement, and appreciation to the staff. We also received letters of appreciation and gifts from citizens and companies. Special allowances were also provided to all employees. We will need to examine how such efforts improve mental health among hospital health‐care workers.

This study has several limitations. First, the present study used the CFA analysis to replicate the factors and their comprising items that were confirmed in the H1N1 influenza pandemic. However, the limitation of conducting CFA by the previous model may dismiss other models that fit more appropriately to the present pandemic. Second, the response rate of the present study was 29.6%. A low response rate can give rise to sampling bias if the nonresponse is unequal among the participants. However, the results of this study were consistent with those of previous studies and seemed to reflect the mental health of hospital health‐care workers. Third, we did not assess other common mental health problems, such as depression. However, the association between IES and depression has been pointed out in previous studies^38^; thus, the IES‐R scores may accurately reflect the mental health of the staff. Fourth, as our study was conducted at a single health‐care facility, its external validity is limited. These results, however, are similar to those of another study conducted in an urban health‐care setting in Japan.^28^ The study showed that female nurses, when compared with doctors who were low‐risk workers, and people aged between 40 and 49 years who were high‐risk workers experienced more event‐related distress. This finding, which supports our analysis, suggests that our result has some degree of external validity.

## AUTHOR CONTRIBUTIONS

Haruko Fukushima, Hissei Imai, Chisato Miyakoshi, Hiroyuki Miyai, Kyohei Otani, Shinsuke Aoyama, and Kunitaka Matsuishi were involved in study design. Hissei Imai and Chisato Miyakoshi contributed to data analysis. Haruko Fukushima, Hiroyuki Miyai, Kyohei Otani, and Kunitaka Matsuishi contributed to the acquisition of data. Haruko Fukushima drafted the initial manuscript, which was then revised by Hissei Imai, Chisato Miyakoshi, and Kunitaka Matsuishi. All authors approved the final manuscript.

## CONFLICTS OF INTEREST

The authors declare no conflicts of interest.

## ETHICS APPROVAL STATEMENT

The study protocol was approved by the Ethics Committee of Kobe City Medical Center General Hospital (no. zn200726). Written informed consent was obtained from the participants.

## Data Availability

The dataset supporting the conclusion of the current study is available from the corresponding author on reasonable request.
